# Phloem development in nematode-induced feeding sites: the implications of auxin and cytokinin

**DOI:** 10.3389/fpls.2013.00241

**Published:** 2013-07-08

**Authors:** Birgit Absmanner, Ruth Stadler, Ulrich Z. Hammes

**Affiliations:** ^1^Cell biology and plant biochemistry, University RegensburgRegensburg, Germany; ^2^Molecular Plant Physiology, Friedrich Alexander University Erlangen NürnbergErlangen, Germany

**Keywords:** sieve element, companion cell, protophloem, metaphloem, root knot nematode, cyst nematode

## Abstract

Sedentary plant parasitic nematodes such as root-knot nematodes and cyst nematodes induce giant cells or syncytia, respectively, in their host plant's roots. These highly specialized structures serve as feeding sites from which exclusively the nematodes withdraw nutrients. While giant cells are symplastically isolated and obtain assimilates by transporter-mediated processes syncytia are massively connected to the phloem by plasmodesmata. To support the feeding sites and the nematode during their development, phloem is induced around syncytia and giant cells. In the case of syncytia the unloading phloem consists of sieve elements and companion cells and in the case of root knots it consists exclusively of sieve elements. We applied immunohistochemistry to identify the cells within the developing phloem that responded to auxin and cytokinin. Both feeding sites themselves did not respond to either hormone. We were able to show that in root knots an auxin response precedes the differentiation of these auxin responsive cells into phloem elements. This process appears to be independent of B-type Arabidopsis response regulators. Using additional markers for tissue identity we provide evidence that around giant cells protophloem is formed and proliferates dramatically. In contrast, the phloem around syncytia responded to both hormones. The presence of companion cells as well as hormone-responsive sieve elements suggests that metaphloem development occurs. The implication of auxin and cytokinin in the further development of the metaphloem is discussed.

## Introduction

Nematodes are destructive pathogens that inflict considerable damage to a wide variety of plants (Sasser and Freckman, 1987). Among the plant-parasitic nematodes are migratory species and sedentary nematodes. The latter invade a host plant's root and induce specialized feeding sites, from which exclusively nutrients are withdrawn. The two most prominent groups of sedentary nematodes are root knot nematodes (RKN) and cyst nematodes (CN). The life cycles of these two types of nematodes are fairly similar, however, there are also marked differences. The life cycle starts when the juveniles hatch from the eggs. The only free-living larval stage is the second juvenile (J2) stage, which migrates through the soil in search for a host plant. The juveniles enter the root and move toward the stele where they set up the feeding site. How the initial cell is chosen is not clear, but after injection of secretions from subpharingeal glands the initial cell undergoes marked changes that differ considerably between RKN and CN. The precise mechanisms that lead to the differentiation processes of both feeding sites are complex but remain poorly understood [for a recent review see Gheysen and Mitchum (2011)]. While the initial events have received much attention, the later stages of a successful interaction between sedentary nematodes and plants are only poorly understood. It is known that the development of nematodes is dependent on the nutritional status. If nutrient supply is high more females develop whereas more males develop when nutrient supply is not optimal (Trudgill, 1967, 1972). The male nematodes leave the feeding sites and CN males will then proceed to fertilize females whereas the offspring of RKN is formed by parthenogenesis (Liu et al., 2007). Once the eggs are formed the female dies. The eggs of RKN are disposed in a gelatinous egg-mass and in case of cyst nematodes the eggs are embedded in the eponymous cyst, which is formed by the dead female and represents a durable stage that can survive in the soil for years.

The events on the plant side that lead to a successful support of the different feeding sites share some similarities, i.e., induction, growth of the feeding site and vascularization but there are also marked differences.

In the case of RKN there are typically four to eight founder cells that eventually develop into giant cells (GCs) (Jones and Payne, 1978). These cells enlarge dramatically and can reach sizes of up to a millimeter. Throughout their entire lifetime GCs retain single cell identity. Both GCs and the surrounding tissue give rise to the eponymous root knot that can be easily spotted by the naked eye. Interestingly, the size of the root knot does not correlate strictly with GC size or the amount of other tissues in the structure. It rather appears to be specific for a particular interaction. For example, *Meloidogyne artiellia* induces huge root-knots on chickpea (*Cicer arientium*) but only small ones on faba bean (*Vicia faba*), whereas in the case of *Meloidogyne incognita* the situation is the opposite (Vovlas et al., 2005). In the mature stage a high metabolism, a large density of organelles, multiple nuclei, the lack of a large central vacuole and marked cell wall ingrowths characterize fully developed giant cells (Jones and Northcote, 1972). The latter hallmark is typically found also in other transfer cells which are characterized by elevated transport over their plasma membrane to facilitate the development of sink tissues, e.g. seeds (Offler et al., 2002). As it is the case for transfer cells in seed coats, it could be shown that GCs are symplastically isolated from the surrounding tissue, i.e., not connected by plasmodesmata (Hoth et al., 2008). Consistent with the symplastic isolation and their identity as transfer cells, transport proteins are massively regulated during the infection and loss of transporter function leads to severe phenotypes in nematode development (Hammes et al., 2005; Marella et al., 2013). To support the growth of the giant cells and, consequently, the nematodes, the vasculature surrounding the GCs changes significantly. Xylem vessels appear to be distorted and often they are not connected to each other (Fester et al., 2008). The phloem undergoes even more dramatic changes. Companion cells (CCs) are initially present around developing GCs but they appear to be absent from the phloem in the mature stages (Hoth et al., 2008). Whether CCs lose their identity, are consumed during the development or they de-differentiate remains elusive. The phloem in a mature root knot consists exclusively of cells that display sieve element (SE) characteristics and often remain nucleated. Furthermore, these cells are massively connected to each other by plasmodesmata to facilitate assimilate flow from cell to cell (Hoth et al., 2008). How the assimilates get out of the phloem and into the GC apoplast is not known at present.

In the case of CN the initial founder cell breaks down the cell walls to the neighboring cells, which eventually leads to the formation of a syncytium (Sijmons et al., 1991; Golinowski et al., 1996). In its mature stage the syncytium is functionally equivalent to the giant cells, again sharing very obvious similarities such as the dense cytoplasm, multiple nuclei and the high metabolic activity of a multicellular entity. Striking differences can be observed in terms of vascularization and nutrient flow into the syncytium. In both types of feeding structures new phloem is formed. However, in contrast to the situation in root knots, sieve elements and companion cells are present in this phloem. Nevertheless, both cell types are induced around syncytia to facilitate nutrient supply (Hoth et al., 2005). The phloem of syncytia, too, is massively interconnected by plasmodesmata but in contrast to giant cells syncytia become connected to the phloem by secondary plasmodesmata (Hoth et al., 2008). As a consequence, the flow of assimilates into syncytia occurs passively by diffusion through plasmodesmata. Consistent with symplastic loading of nutrients into syncytia, transport proteins play a far less important role (Puthoff et al., 2003).

In the last couple of years enormous progress has been made to identify and understand the molecular players and the precise mechanisms that define tissue identity in the vasculature under “normal” circumstances reviewed by (Miyashima et al., 2013). In the primary root meristem phloem and xylem development are closely linked. A mutant in the *ALTERED PHLOEM DEVELOPMENT (APL)* gene fails to specify phloem and in its place xylem development occurs. It is also known that the combinatorial effects of the two phytohormones auxin and cytokinin play a crucial role in specifying the xylem axis, thereby defining the spatial constraints in which phloem development can take place (Bishopp et al., 2011a,b,c). On the other hand, surprisingly little is known about the events that are associated with “secondary” vascularization events, i.e., the connection of tissues or organs to the existing vasculature or its secondary proliferation. Events similar to the vascularization of nematode-induced feeding sites described above are also observed in a number of other pathogen interactions, for example crown galls induced by *Agrobacterium tumefaciens* or the interaction with parasitic plants such as dodder (*Cuscuta sp*.) or mistletoe (*Viscum sp*.) (Aloni et al., 1995; Birschwilks et al., 2006). Furthermore, the nodules, which are formed during the beneficial interaction between nitrogen fixing bacteria and legumes, are vascularized to maintain the exchange of fixation products and assimilates, respectively (Schultze and Kondorosi, 1998). Vascularization occurs not only in organismic interactions: plants are also able to form new vascular elements after wounding or grafting (Behnke and Schulz, 1980; Kollmann and Glockmann, 1985; Rachowbrandt and Kollmann, 1992). In the latter case, which is of great agronomic relevance, even vascular elements of different species become connected to support healthy growth of the chimaera. In all these processes the phytohormone auxin seems to play an important role, often also in combination with other phytohormones (Aloni, 1979; Aloni et al., 1990, 1995). In the case of nematode infection the early events of feeding site initiation were shown to be dependent on auxin (Goverse et al., 2000; Karczmarek et al., 2004; Grunewald et al., 2009). On the other hand nothing is known about the role of auxin during the vascularization of nematode-induced feeding sites, which occurs at later stages of feeding site development. For this reason we examined the auxin response of the vasculature of wild type Arabidopsis plants and mutants of the auxin signaling pathway throughout the infection with the root knot nematode *Meloidogyne incognita* or the cyst nematode *Heterodera schachtii*. We show that in root knots an auxin response, but no cytokinin response, occurs in cells, which develop into phloem cells. In the mature stage the sieve element marker antibody RS6 (Khan et al., 2007) can label virtually all cells that were auxin-responsive. Using GUS staining we demonstrate that the corresponding gene is expressed in the phloem cells throughout development. Additionally, the *APL* gene, which is used as a marker for protophloem and companion cells is expressed in these cells. In contrast to the phloem formation around giant cell, the developing sieve elements surrounding syncytia responded to both, auxin and cytokinin but the companion cells responded only to auxin. The data suggest that the phloem around giant cells has protophloem identity and the phloem around syncytia metaphloem identity.

## Materials and methods

### Plant material

Transgenic *Arabidopsis thaliana* lines containing *P*_*DR5*_*:GFP* (Ottenschlager et al., 2003) or *P*_*DR5*_*:GUS* (Ulmasov et al., 1999) were used to monitor auxin response. Cytokinin response was followed using the *P*_*TCS*_*:GFP* line described by (Müller and Sheen, 2008). The *P*_*SUC2*_*:GFP* transgenic line expressing soluble GFP under control of the companion cell specific AtSUC2 promoter was described in (Imlau et al., 1999). APL promoter activity was monitored in a *P*_*APL*_*:GUS* transgenic line (Bonke et al., 2003). In order to monitor promoter activity of the sieve element marker RS6 a 998 kb fragment upstream of the RS6 coding region was amplified by PCR from Col-0 genomic DNA and introduced into the pMDC162 vector (Curtis and Grossniklaus, 2003) by gateway cloning. Primer sequences are available upon request. The auxin resistant mutant axr1-3/axr4-2 (Hobbie and Estelle, 1995) was obtained from ABRC. The homozygous T-DNA insertion line aux1 was previously published (Yang et al., 2006).

### Growth conditions and infection with nematodes

For surface sterilization, seeds of the lines described above were incubated in 70% ethanol p.A. for 3 min and subsequently in an aqueous solution of 1% NaOCl and 0.1% Mucasol® for 2 min. The seeds were washed 4 times in sterile water, resuspended in 0.1% agarose and plated on Gamborg medium supplied with 2% sucrose. After 2 days of vernalization at 4°C, plates were incubated in a growth chamber under short day conditions at 22°C. Two weeks later, the plants were infected with the *H. schachtii* or *M. incognita second stage juveniles (J2)*, respectively. For infection with *H. schachtii*, cysts were harvested from sterile agar stocks and incubated in 3 mM ZnCl_2_ at 25°C. After 4 days, freshly hatched (J2) were collected, washed with sterile water and used for inoculation of plant cultures. In case of *M. incognita*, freshly hatched J2 were obtained from eggs that were collected from infected tomato plants (*Solanum lycopersicum* cv. Moneymaker). The J2 were surface sterilized by incubating them in 0.02% HgCl_2_ for 30 s. Afterwards, they were washed with sterile water and used for inoculation.

### Confocal laser scanning microscopy

GFP fluorescence was analyzed using a Zeiss Axiovert 200 M microscope equipped with a confocal laser scanning unit LSM 510 META using the 488 nm line of the argon laser for excitation and a BP 505–550 filter for selective GFP detection.

### Section preparation and immunohistochemistry

Immunohistchemistry was performed as described (Meyer et al., 2000). Semi-thin-sections with a thickness of 3 μm were prepared at a microtome and were treated with antibodies diluted in blocking buffer as follows: mouse monoclonal RS6 antibody 1:10 (Khan et al., 2007); rabbit polyclonal anti-GFP antiserum 1:100; goat anti-mouse-Cy3 conjugate 1:80 (Dianova); goat anti-rabbit-Cy2 conjugate 1:80 (Dianova). Sections were mounted in 50% glycerol and fluorescence was analyzed at an Axioskop FL epifluorescence microscope (Zeiss) using the Zeiss filter sets no. 46 (Cy2, excitation: BP 500/20, beam splitter: FT 515, emission: BP 535/30) and no. 15 (Cy3, excitation: BP 546/12, beam splitter: FT 580, emission: LP 590).

### Staining for GUS activity

For histochemical localization of GUS activity, root material was infiltrated with 1.25 mM X-Gluc in staining solution (0.05 M NaPO_4_ pH 7.2, 0.5 mM K_3_Fe(CN)_6_, 0.5 mM K_4_Fe(CN)_6_, 1% Triton X-100) and incubated at 37°C until blue staining was clearly visible. Samples were washed three times with 0.05 M NaPO_4_ pH 7.2 and analyzed by brightfield microscopy. For thin sections, root material was embedded in 5% low melting agarose and sectioned to 60 μm using a vibratome.

## Results

We wanted to investigate the effect of auxin and cytokinin on phloem development in the two different feeding sites, root knots and syncytia induced by RKN or CN, respectively. The importance of auxin during the very early stages of nematode infestation has been documented. In order to investigate which cells within the feeding sites show an auxin response we used the well-documented *P*_*DR*_*5:ER-GFP* plant line that expresses ER-localized GFP under control of the auxin responsive DR5 promoter (Ottenschlager et al., 2003).

### Auxin response in uninfected wild type roots

**Figure 1:**
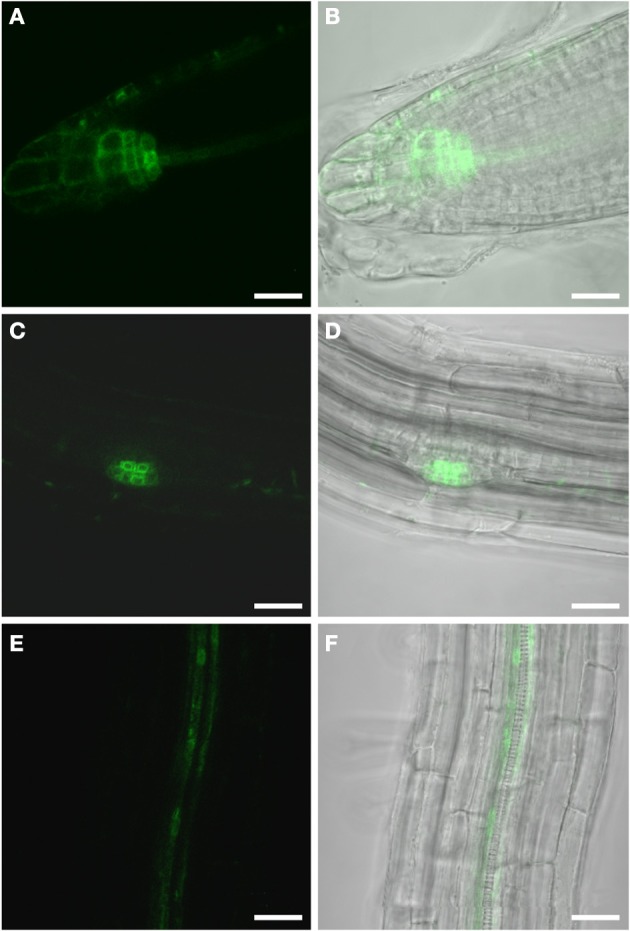
**GFP fluorescence in uninfected roots of 2-week old *P*_*DR5*_*:ER-GFP* Arabidopsis plants.** Fluorescence is observed in auxin responsive tissues. **(A,B)** Root tip **(A)** GFP channel **(B)** Overlay of GFP fluorescence and bright field image. **(C,D)** Lateral root primordium **(C)** GFP channel **(D)** Overlay of GFP fluorescence and bright field image. Within the vasculature approximately 2 cm above the root tip **(E,F)** fluorescence is observed in two cell files. **(E)** GFP fluorescence **(F)** Overlay of GFP fluorescence and bright field image. Scale bars represent 20 μm.

**Figure 2:**
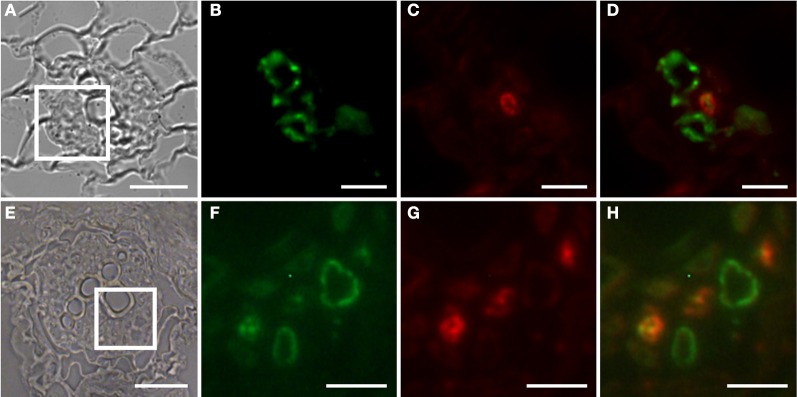
**Identification of auxin responsive cells by immunohistochemistry in sections through roots of 2-week old *P*_*DR5*_*:ER-GFP* Arabidopsis plants. (A–D)** Section through a root approximately 2 cm above the root tip corresponding to the part of the root in which early giant cell development occurs. **(E–H)** Section through a slightly older part of the root. **(B–D)** and **(F–H)** are details of **(A)** and **(E)**, respectively, indicated by the square. **(B,F)** Auxin responsive cells decorated by a GFP antibody. The position is consistent with a companion cell identity. Green color results from a Cy2-conjugated secondary antibody. **(C,G)** Cells decorated with the sieve element marker antiserum RS6. Red color results from a Cy3-conjugated secondary antibody. **(D,H)** are overlays of **(B,C)** and of **(F,G)**, respectively. Scale bars represent 20 μm in **(A,E)** and 5 μm in **(B)**–**(D)** and **(F)**–**(H)**.

The Arabidopsis plants used for infection were grown on Gamborg medium for 2 weeks prior to infection (see materials and methods section). We examined GFP fluorescence in uninfected control roots of this age (Figure 1). In agreement with previous data fluorescence was observed in the root tips (Figures 1A,B) and in lateral root primordial (Figures 1C,D). In the differentiated parts of the root, approximately 2 cm distal of the root tip, fluorescence was also always detectable in two cell files in the stele (Figures 1E,F). In order to identify the cell type that displayed the auxin response within the stele we performed immunolocalization experiments on sectioned *P*_*DR*_*5:ER-GFP* plant root material (Figure 2). We used the previously described monoclonal RS6 antiserum to identify sieve elements (Khan et al., 2007) and a polyclonal GFP antiserum to identify the auxin responsive cells. In the stele approximately 2 cm distal of the root tip the auxin response, shown in green, occurred in cells directly adjacent to the sieve elements, shown in red (Figures 2B–D). Cells decorated by the antisera were obviously located at the phloem pole of the stele. Based on the characteristic position of the auxin responsive cells with respect to the sieve elements and the xylem, cells with wide lumens and thick walls, we identified these cells as companion cells. In slightly older sections of the root (approximately 3 cm distal of the tip) the pattern observed was similar (Figures 2E–H). The auxin responsive cells were always directly adjacent to the sieve elements that were labeled by the RS6 antiserum. Taken together, these data show that a constitutive auxin response occurs in cells within the fully differentiated phloem of 2-week-old Arabidopsis wild type roots. The auxin response occurs in cell directly adjacent to sieve elements, the companion cells.

### Auxin response in root knots induced by *meloidogyne incognita*

**Figure 3:**
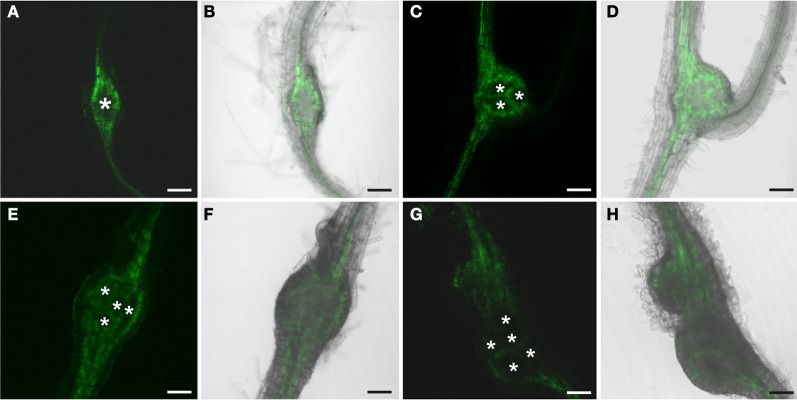
**GFP fluorescence in roots of *P*_*DR5*_*:ER-GFP* Arabidopsis plants infected with *M. incognita*.** Fluorescence is observed in auxin responsive tissues. Note the net-like structure of the tissue consisting of cells displaying GFP fluorescence. **(A**,**C,E,G)** GFP fluorescence. **(B**,**D**,**F,H)** Overlay of GFP fluorescence and bright field image. **(A,B)** 7 dai, **(C,D)** 14 dai **(E,F)** 17 dai, **(G,H)** 21 dai. Scale bars represent 100 μm.

**Figure 4:**
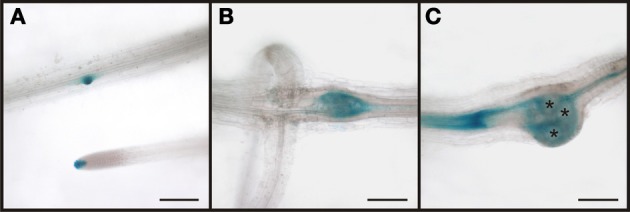
**Auxin response visualized by GUS-staining in *M. incognita* infected *P*_*DR5*_*:GUS* plant roots. (A)** uninfected parts of the root displayed a normal staining pattern. The tissue within the root-knot 7 dai **(B)** and 17 dai **(C)** displayed an auxin response. Giant cells (asterisks in **C**) were not stained. Scale bars represent 200 μm.

**Figure 5:**
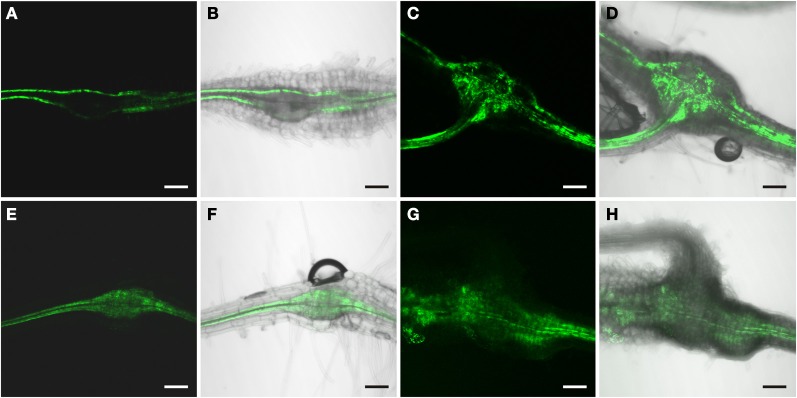
**Comparison of GFP fluorescence in root-knots of a *M. incognita* infected *P*_*SUC2*_*:GFP* plant (A–D) and a *P*_*DR5*_*:ER-GFP* plant (E–H) at 7 dai (A and B, E and F) and 14 dai (C and D, G and H).** At 7 dai, GFP-fluorescence is limited to the sieve element/companion cell complex of the regular phloem in **(A**,**B)** but present in more cells in **(E,F)**. At 14 dai, GFP fluorescence is seen in a net-like pattern in both a *P*_*SUC2*_*:GFP* **(C,D)** and a *P*_*DR5*_*:ER-GFP* plant **(G,H)**. **(A,C,E,G)** GFP fluorescence, **(B,D,F,H)** Overlay of GFP fluorescence and bright field image. Scale bars represent 100 μm.

**Figure 6:**
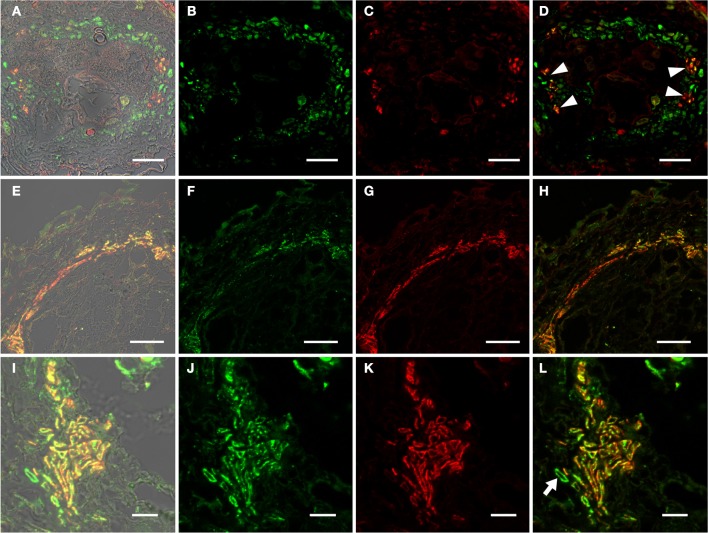
**Identification of auxin responsive cells by immunohistochemistry in sections through root-knots of *P*_*DR5*_*:ER-GFP* Arabidopsis plants infected with *M. incognita*. (A–D)** Section through a root knot 10 dai. **(E–H)** Section through root knot 17 dai. **(I–L)** detail of a section through a root knot 17 dai. **(B,F,J)** Auxin responsive cells decorated by a GFP antibody. Green color results from a Cy2-conjugated secondary antibody. **(C,G,K)** Cells decorated with the sieve element marker antiserum RS6. Red color results from a Cy3-conjugated secondary antibody. **(D**,**H,L)** are overlays of (**B**,**C)**, **(F,G)**, and **(J,K)**, respectively. Arrow heads in **(D)** point to RS6-positive cells located at two poles within the stele, probably the location of the initial phloem poles. Arrow in **(L)** points to the rarely observed cells that are exclusively labeled by the GFP antiserum. Scale bars represent 20 μm in **(A**–**H)** and 10 μm in **(I**–**L)**.

In order to identify auxin responsive cells in root knots of *P*_*DR*_*5:ER-GFP* plants we monitored GFP-fluorescence throughout their development (Figure 3). Above and below young galls, approximately 7 days after infection (dai), fluorescence was visible in the cell files described above (Figures 3A,B). Within the root knot fluorescence was observed surrounding an area that displayed no fluorescence (asterisk in A). Approximately 14 dai (Figures 3C,D) and 17 dai (Figures 3E,F) fluorescence was observed in a net-like pattern that surrounded fluorescence-free areas, which are in the position of the giant cells (asterisks). In fully mature and differentiated root knots the fluorescence pattern was similar but in general slightly weaker (Figures 3G,H). We never observed fluorescence at the position of giant cells using *P*_*DR5*_*:ER-GFP* plants. To increase the sensitivity we also infected *P*_*DR5*_*:GUS* plants (Figure 4). The data obtained supported the notion that an auxin response occurs in the root knot but is absent from the giant cells, which are readily visible (asterisks in Figure 4C). The fluorescence or staining pattern observed throughout development of the root knot was highly reminiscent of that described in a previous study in which the fluorescence resulting from GFP expressed under control of the companion cell specific *SUC2* promoter diffused from the companion cells into the sieve elements surrounding the giant cells (Hoth et al., 2008). In order to determine the earliest time point at which the net-like pattern becomes apparent first, we directly compared the fluorescence pattern of *P*_*DR5*_*:ER-GFP* and *P*_*SUC2*_*:GFP* plants 7 dai and 14 dai (Figure 5). At 7 dai GFP fluorescence of *P*_*SUC2*_*:GFP* plants was still limited to the companion cells of the vasculature, indicating that plasmodesmata allowing the diffusion of GFP had not formed (Figures 5A,B). In contrast, in *P*_*DR5*_*:ER-GFP* plants the occurrence of the fluorescence was already visible (Figures 5E,F). At 14 dai the plasmodesmata allowing the diffusion of GFP from the companion cells into the cells surrounding the giant cells as described earlier had occurred (Figures 5C,D) (Hoth et al., 2008). The pattern of fluorescence observed in *P*_*DR5*_*:ER-GFP* plants looked strikingly similar the that of *P*_*SUC2*_*:GFP* plants(Figures 5G,H). These data indicate that the cells that will eventually form the net surrounding giant cells experience an auxin response prior to their differentiation and also prior to becoming interconnected by plasmodesmata. To demonstrate this at the cellular level we performed immunolocalization experiments using the GFP antiserum to identify cells responding to auxin and the RS6 antiserum to decorate sieve elements (Figure 6). Figures 6A–D shows a section through a root-knot at 10 dai. The giant cells are readily identified. At two opposite poles an elevated number of cells that were decorated with the RS6 serum, shown in red, had formed (Figure 6C; arrow heads in Figure 6D). Most cells that were adjacent to the giant cells were labeled by the GFP antiserum, shown in green, thereby forming a closed ring surrounding the giant cells (Figure 6B). Figures 6E–L shows that at 17 dai the giant cells become surrounded by a high number of sieve elements, decorated by the RS6 antiserum (Figures 6G,H), consistent with previous findings (Hoth et al., 2008). Surprisingly, almost all cells that were labeled with the sieve element marker were also labeled by the GFP antiserum (Figures 6H,L). This indicates that all the cells that differentiated into sieve elements were experiencing an auxin response and that very early during the infection cycle the cells surrounding the giant cells become auxin responsive and after the onset of this auxin response differentiate into sieve elements. We hardly ever observed cells that were labeled by the GFP serum exclusively. One rare example is shown in Figure 6L (arrow). This suggests that at 17 dai the formation of the sieve element net is complete.

It is noteworthy that we never detected GFP in giant cells, which means that these cells themselves do not respond to auxin.

**Figure 7:**
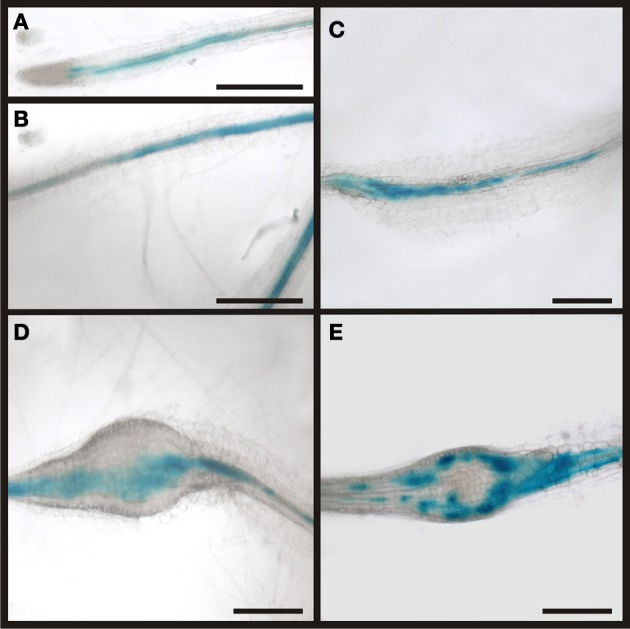
**Activity of the RS6 promoter visualized by GUS staining in *P*_*RS6*_*:GUS* plants.** In control sections of plants roots infected with *M. incognita* staining is observed throughout the stele **(A,B)**. In root knots 5 dai the staining is still limited to the stele **(C)**. In root-knots 10 dai the staining is spreading out but still in the position of the original phloem pole **(D)**. In mature root-knots **(E)**, 17 dai, staining is observed around the giant cells. Scale bars represent 200 μm.

In order to demonstrate that the RS6 promoter is active throughout the development of the root knot we performed GUS staining (Figure 7). We could show that the expression pattern of the RS6 promoter mirrors the formation of the symplastic domain specified by the diffusion of GFP. In uninfected roots of infected plants staining was limited to the stele in two cell files (Figures 7A,B). The staining pattern was sustained in early developmental stages of root knot development (Figure 7C). At 10 dai staining became diffuse and extended from the phloem poles (Figure 7D). In mature root knots staining was observed in a net-like pattern surrounding the giant cells. This indicates that once cells become auxin responsive the RS6 gene is transcribed.

**Figure 8:**
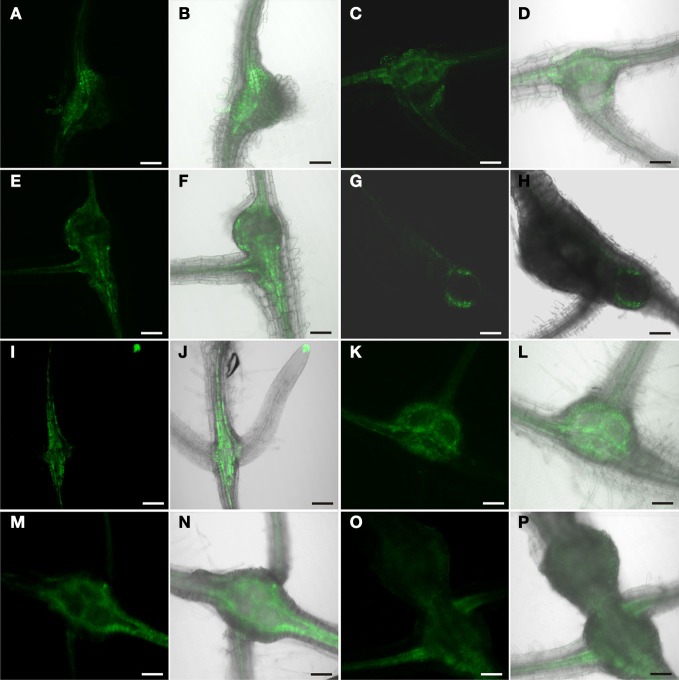
**GFP fluorescence in *M. incognita*-induced root knots of plants expressing ER-GFP under control of the DR5 promoter in a mutant background. (A**–**H)** aux1 background, **(I–P)** axr1-3/axr4-2 background. Fluorescence is observed in auxin responsive tissues in a seemingly unaltered pattern. **(A**,**C,E,G,I,K,M,O)** GFP fluorescence. **(B,D,F,H,J,L,N,P)** Overlay of GFP fluorescence and bright field image. **(A,B,I,J)** 7 dai, **(C,D,K,L)** 14 dai (**E**,**F**,**M,N)** 17 dai, **(G,H,O,P**) 21 dai. Scale bars represent 100 μm.

In order to address the role of auxin in phloem development in root knots further we employed auxin resistant or auxin transport mutants. Unfortunately, strong auxin response mutants or auxin resistant mutants display severely disturbed root development and a highly reduced number of lateral roots, which are missing entirely in some cases. For this reason we used auxin mutants for which it was reported that they can be infected with nematodes (Goverse et al., 2000). The aux1 mutant is a knock-out line carrying a T-DNA insertion in the gene encoding the high-affinity auxin influx carrier AUX1 (Yang et al., 2006); the axr1-3/axr4-2 mutant displays a high degree of auxin resistance (Hobbie and Estelle, 1995). The DR5 marker was introgressed into the mutant backgrounds. Figure 8 shows the auxin response in aux1 mutants (Figures 8A–H) and axr1-3/axr4-2 double mutants (Figures 8I–P). The fluorescence pattern observed was essentially undistinguishable from that of wild type plants. Furthermore, the females on these plants developed properly. Thus, the auxin response leading to phloem differentiation occurred normally in these mutants. This indicated that phloem development is independent of AUX1-mediated auxin influx as well as of AXR1 action. However, it is noteworthy that we observed numerous lateral roots associated with the root knots (Figures 8D,F,H,J,L,N,P) despite the fact that the mutants show strongly reduced lateral root formation (Hobbie and Estelle, 1995). Also, this is somewhat in contrast to the observation of (Goverse et al., 2000) who described that upon infection of the axr1-3/axr4-2 mutant with *H. schachtii*, no lateral roots at all were found associated with feeding sites.

### Auxin response in the phloem adjacent to syncytia induced by *heterodera schachtii*

**Figure 9:**
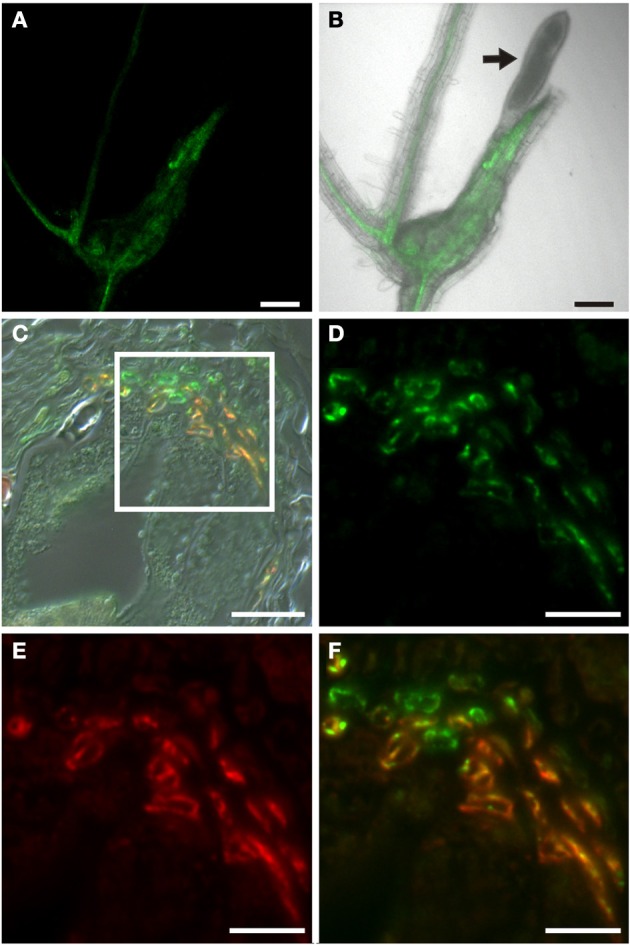
**Auxin response in roots of *H. schachtii*-infected *P*_*DR5*_*:ER-GFP* Arabidopsis plants 14 dai. (A,B)** GFP fluorescence in intact syncytia. **(A)** GFP fluorescence, **(B)** Overlay of GFP fluorescence and bright field image, arrow points to the nematode. **(C)** Bright field image of a section through a syncytium 14 dai. **(D–F)** is a detail of the indicated position in **(C)**. (**D)** Auxin responsive cells decorated by a GFP antibody. Green color results from a Cy2-conjugated secondary antibody. **(E)** Cells decorated with the sieve element marker antiserum RS6. Red color results from a Cy3-conjugated secondary antibody. **(F)** is an overlay of **(D,E)**. Scale bars represent 100 μm in **(A,B)**, 20 μm in **(C)** and 10 μm in **(D**–**F)**.

In order to identify auxin-responsive cells in the tissues adjacent to the syncytium and to understand if the syncytium itself responds to auxin in its mature stage we infected *P*_*DR5*_*:ER-GFP* plants with J2 juveniles of the beet-cyst nematode *Heterodera schachtii*. The fluorescence pattern observed was also in this case very much reminiscent of that when using *P*_*SUC2*_*:GFP* plants (Hoth et al., 2005, 2008). However, the syncytium itself appeared to be free of fluorescence (Figures 9A,B). To identify the cells that responded to auxin we performed immunohistochemistry using the GFP antiserum to identify auxin-responsive cells and the RS6 antiserum to label sieve elements (Figures 9C–F). We were able to identify cells that had differentiated into sieve elements at an elevated number (Figure 9E) consistent with earlier studies (Hoth et al., 2005). Again, most cells that were decorated by the RS6 antiserum were also labeled by the GFP antiserum (Figure 9D), suggesting that also the sieve elements around syncytia or the cells that they developed from, were responding to auxin. In marked contrast to the situation in root knots we also found cells which were clearly labeled only by the GFP antiserum (Figure 9F). The characteristic position of these cells with respect to the sieve elements as well as to the syncytium is strongly suggesting that these cells are companion cells, which were clearly identified previously (Hoth et al., 2005). As it was the case for giant cells we never observed a GFP-positive syncytium using this method. This indicates that both feeding sites don't display an auxin response in their mature and fully differentiated stage.

Taken together, the results indicate that in both nematode interactions cells in vicinity of the feeding site are exposed to an auxin response. In the case of root knots this leads to the development of sieve elements and in the case of the syncytia-associated phloem to the proliferation of companion cells and sieve elements. This differential response cannot be explained by the auxin responsiveness alone.

### Cytokinin response in root knots

**Figure 10:**
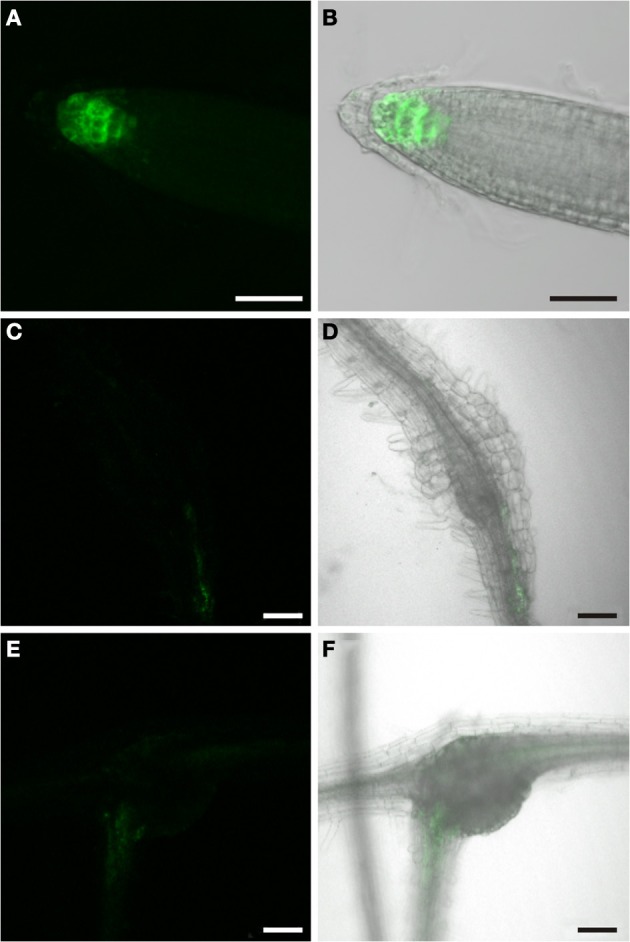
**GFP fluorescence in Arabidopsis plants expressing ER-GFP under the control of the TCS-promoter.** Fluorescence is observed in cytokinin responsive tissues. **(A,B)** GFP fluorescence in the root tip of a 2 week old *P*_*TCS*_*:ER-GFP* plant. **(A)** GFP fluorescence. **(B)** Overlay of GFP fluorescence and bright field image. **(C,D,E,F)** GFP fluorescence in *M. incognita*–induced root-knots in *P*_*TCS*_*:ER-GFP* plants at 7 dai **(C,D)** and 14 dai **(E,F)**. Throughout development staining was diffuse and virtually absent from root knots. **(C,E)** GFP fluorescence. **(D,F)** Overlay of GFP fluorescence and bright field image. Scale bars represent 50 μm in **(A,B)** and 100 μm in **(C**–**F)**.

Auxin is well-known to act in a finely tuned combination with other phytohormones, particularly cytokinin. In order to study the cytokinin response we used *P*_*TCS*_*:ER-GFP* Arabidopsis plants that express ER-localized GFP under control of a synthetic cytokinin-responsive promoter. The TCS promoter contains the concatemerized binding motifs for B-type response regulators which in Arabidopsis mediate the transcriptional activation of early cytokinin target genes, fused to a minimal CamV35S promoter (Müller and Sheen, 2008). Figure 10 shows the typical fluorescence observed in these plants. Cytokinin response was seen in the root tip (Figure 10A), consistent with prior studies (Müller and Sheen, 2008). In contrast to the findings for the *P*_*DR5*_*:ER-GFP* plants we did not observe fluorescence in the stele, indicating that companion cells do not display a marked cytokinin response. Interestingly, we also never detected a cytokinin response in root knots neither in the giant cells themselves nor the surrounding tissues throughout the entire development (Figures 10C–F). This suggests that in the case of the phloem development in root knots auxin seems to play a crucial role and cytokinin response mediated by B-type RR genes does not occur at a level that can be detected using this approach.

### Cytokinin response of syncytia-adjacent phloem

**Figure 11:**
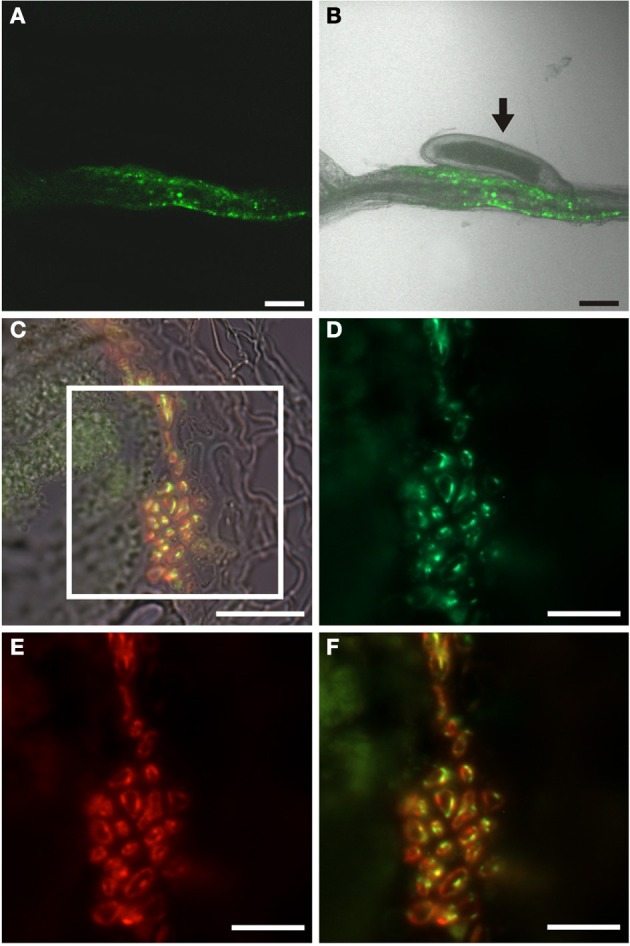
**Cytokinin response in roots of *H. schachtii*-infected *P*_*TCS*_*:ER-GFP* Arabidopsis plants 14 dai. (A,B)** GFP fluorescence in intact syncytia. **(A)** GFP fluorescence, **(B)** Overlay of GFP fluorescence and bright field image, arrow points to the nematode. **(C)** Bright field image of a section through a syncytium 14 dai. **(D–F)** is a detail of the indicated position in **(C)**. **(D)** Cytokinin responsive cells decorated by a GFP antibody. Green color results from a Cy2-conjugated secondary antibody. **(E)** Cells decorated with the sieve element marker antiserum RS6. Red color results from a Cy3-conjugated secondary antibody. **(F)** is an overlay of **(D,E)**. Scale bars represent 100 μm in **(A,B)**, 20 μm in **(C)** and 10 μm in **(D**–**F)**.

In order to investigate if the phloem around syncytia responded to cytokinin we infected *P*_*TCS*_*:ER-GFP* Arabidopsis plants with *H. schachtii* J2 juveniles. In contrast to the situation in root knots we observed a cytokinin response associated with syncytia (Figures 11A,B). To gain insights at the cellular level we performed immunolocalization of GFP and the RS6 marker (Figures 11C–F). We could demonstrate the presence of GFP in cells associated with the syncytia (Figure 11D). Colocalization with the RS6 marker antibody confirmed that these cells were sieve elements. Interestingly—and in marked contrast to the pattern observed for the auxin response—we failed to detect cells that were labeled by the GFP antibody exclusively. This indicates that also the companion cells associated with the syncytia do not display a cytokinin response. The phloem surrounding syncytia is made up of companion cells, which display the same response to hormones as companion cells in uninfected roots, and of sieve elements which are responsive to both auxin and cytokinin. The fact that the sieve elements respond to the hormones clearly points to the notion that also these sieve elements were formed *de novo* and either still contain nuclei or lost them very shortly before.

### Identity of the phloem around giant cells

**Figure 12:**
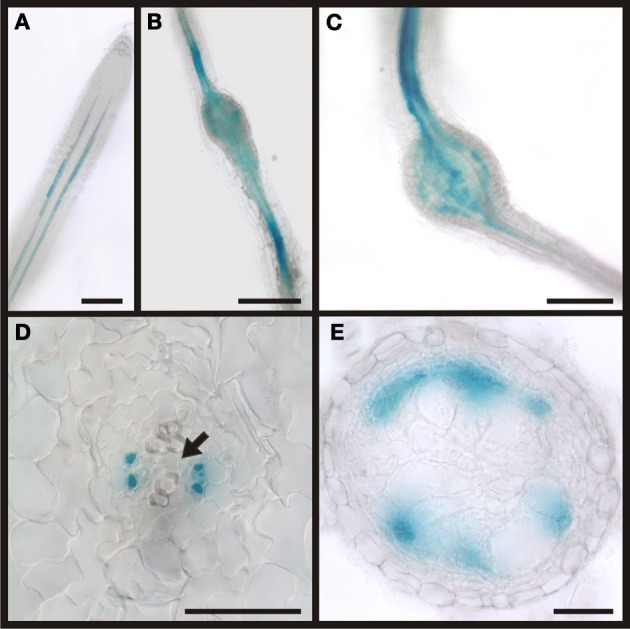
**Activity of the APL promoter visualized by GUS staining in *P*_*APL*_*:GUS* plants.** In control sections of plants roots infected with *M. incognita* staining is observed in two cell files within the stele (**A)**. In root knots 7 dai the staining is limited to the stele but also observed in patches within the root knot **(B)**. In mature root knots 17 dai staining is observed in a net-like pattern in cells surrounding the giant cells **(C)**. **(D,E)** Cross sections through a root knot 17 dai. **(D)** section through the very beginning of the root knot indicated by the separation of the xylem elements (arrow). GUS staining is limited to four cells, probably companion cells. **(E)** Cross section through the center of the root knot. Promoter activity indicated by the GUS staining is observed in the cells surrounding giant cells. Scale bars represent 100 μm in **(A)**, 200 μm in **(B,C)** and 50 μm in **(D,E)**.

The phloem around giant cells is clearly different from the phloem surrounding syncytia and the phloem that is typically found in roots. The most obvious observation is the absence of companion cells and the fact that nuclei were observed. In order to gain more insight about the identity of this phloem by infection of *P*_*APL*_*:GUS* plants (Figure 12). The APL gene is expressed in the nucleated protophloem of developing roots and also in companion cells (Bonke et al., 2003). We observed the expression of the GUS gene in two cell files in uninfected root, consistent with previous studies (Figure 12A). In a root knot 7 dai staining was observed throughout the knot, excluding the giant cells (Figure 12B). Also in a mature root knot at 17 dai the staining was visible as a network around the giant cells (Figure 12C). To confirm this finding, cross sections were prepared. Figure 12D shows a section directly above the root knot, indicated by the separation of the xylem elements (arrow). The staining is clearly seen in the companion cells. In the center of the root knot, the staining domain is much broader and is found in populations of cells adjacent to the giant cells (Figure 12E). As companion cells are absent from the root knot, these APL expressing cells most likely represent the nucleate sieve elements of the root knot phloem. We therefore propose that the phloem around the giant cells has protophloem identity.

## Discussion

### Hormone response in differentiated arabidopsis roots

Auxin is a key regulator of plant development. Its role in root development, both embryonic and postembryonic is documented in close to 2000 papers (state spring 2013). The auxin responsive DR5 promoter has been used widely to visualize auxin response in a multitude of tissues in several plant species throughout development. In the Arabidopsis root an auxin maximum is found in the root tip where it plays a critical role in the positioning of the stem cell niche (Miyashima et al., 2013). This auxin maximum is brought about by the concerted action of a number of transport systems including *pin-formed* (PIN) proteins and ABC-type transporters that transport auxin acropetally in the stele toward the root tip (Benjamins and Scheres, 2008). In the differentiation zone auxin—in combination with cytokinin—also plays a critical role in the specification of proto- and metaxylem (Bishopp et al., 2011a). The auxin responsiveness of these cells was visualized in *P*_*DR5*_*:ER-GFP* plants (Bishopp et al., 2011b,c). Cytokinin response in roots was typically visualized by promoter elements from Arabidopsis response regulators (ARR) that respond to cytokinin, fused to GUS or ER-GFP. The position of the cytokinin maximum in the root tip correlates well with that of auxin and the combinatorial role of both hormones in root development is obvious (Bishopp et al., 2011a; Miyashima et al., 2013). Fairly recently the synthetic TCS promoter, harboring the concatemerized B-type Arabidopsis response regulator (ARR)-binding motifs and a minimal 35S promoter was introduced to monitor the positive transcriptional output of cytokinin signaling (Müller and Sheen, 2008). It is important to keep in mind that this promoter does not allow drawing conclusions about A-type ARRs that repress cytokinin signaling in a negative-feedback loop (Müller and Sheen, 2007). In both cases, auxin and cytokinin signaling, the vast majority of the studies deal with root tips 2–5 days after germination while clearly differentiated parts of the root 2 or more weeks after germination have not been examined. We found that the expression of reporters driven by both the DR5 and the TCS promoters in root tips and lateral root primordia was consistent with published results (Ottenschlager et al., 2003; Müller and Sheen, 2008). In addition to these well-known auxin responsive tissues we found that companion cells responded to the hormone (Figure 2). While there is no published support for this observation there are several arguments in favor of companion cells exhibiting a constitutive auxin response. First, the proto- and metaxylem that exhibit an auxin response in the differentiation zone are fully developed, i.e., they are transformed into dead, water conducting vessels and the position of the DR5-positive cells with respect to the sieve elements clearly argues against a possible xylem identity. Second, as mentioned above, it is by now commonly accepted that auxin and cytokinin are transported in the stele, particularly in the phloem (Bishopp et al., 2011c). In the developed parts of the root the phloem consists of fully differentiated sieve elements and companion cells. Mature sieve elements loose their nucleus during maturation and consequently the ability to respond to stimuli by transcriptional changes. Companion cells integrate a variety of environmental stimuli for example the decision to flower takes place in these cells (An et al., 2004). It seems reasonable that the companion cells in the conducting phloem exhibit a constitutive auxin response to monitor auxin concentration along the transport path to integrate hormonal information. On the other hand we failed to observe a positive transcriptional output indicated by the absence of fluorescence in *P*_*TCS*_*:ER-GFP* plants. Whether cytokinin signaling and response do not take place or are not important in companion cells of the metaphloem remains to be established by examining the response of the A-type ARRs that repress cytokinin signaling via a negative feedback loop.

It is noteworthy that even if a constitutive auxin response in companion cells occurs, this is probably not the crucial cue in specifying cell identity because in an extensive study no promoter elements in the SUC2 promoter of the companion cell marker were identified that indicated a regulation by either of the hormones (Schneidereit et al., 2008).

### Hormone response in nematode-induced tissues- the role of the feeding site

The first observation we made was that fully differentiated feeding sites themselves—i.e., giant cells or syncytia—never responded to auxin. Other studies found DR5-driven reporter gene activity in young feeding sites (Goverse et al., 2000; Karczmarek et al., 2004; Grunewald et al., 2009). A model in which the feeding site acts as source of auxin, which is transported into the surrounding tissue to induce differentiation of these cells has been proposed (Grunewald et al., 2009). It is known that an auxin response in the syncytia or giant cells occurs at early stages but this phenomenon is a transient process and we therefore failed to detect auxin response in the feeding sites at the time points under investigation. Nevertheless, our data do not exclude the possibility that feeding sites act as a source of auxin to trigger the developmental effects in the surrounding vasculature. DR5 activity only provides information about auxin response but not about auxin concentration. Unfortunately, the auxin concentrations in the feeding sites as well as in the surrounding cells are not known. Therefore, it is currently impossible to resolve this issue.

With respect to the feeding sites themselves the same observation was made for cytokinin. Neither in syncytia nor in giant cells TCS promoter activity could be detected. It is possible that A-type ARRs are positively regulated in feeding sites, which we would not detect in *P*_*TCS*_*:ER-GFP* plants. However, in a previous study the response of the A-type ARR5 promoter, visualized by GUS staining, was investigated in RKN-infested roots. In agreement with our data also this promoter did not appear to be active in the feeding site itself, indicating that cytokinin response does not occur (Lohar et al., 2004). The same logic as for auxin also applies in the case of cytokinins: it is possible that the feeding site itself does not respond to cytokinin but acts a source of the hormone that triggers changes in the surrounding tissues.

### Hormone response in nematode-induced tissues- what triggers the differentiation of phloem surrounding feeding sites?

Irrespective of the source of the hormones our data indicate that the phloem around syncytia and giant cells responds to auxin and/or cytokinin and that the ratio of auxin to cytokinin is involved in the differential vascularization of both feeding sites. The results obtained for the two different types of vasculature found in RKN and CN induced tissues are discussed separately in the following sections.

#### Root knots

The phloem around giant cells consists of cells that display exclusively sieve element identity but do not express the companion cell marker *SUC2* (Hoth et al., 2008). In the present study we show that the cells surrounding giant cells display an auxin response as early as 1 week after infection. At this time the vasculature surrounding the giant cells appears unaffected. The *SUC2* gene appears to be expressed normally and the formation of plasmodesmata allowing the diffusion of GFP out of the regular root phloem and into the nematode-induced phloem tissue and there from cell to cell that is found in later stages of RK development obviously has not occurred (Figures 5A,B). Between 1 and 2 weeks after infection the cells change their identity. The sieve element marker RS6 is expressed and readily detected (Figures 6A–D) and the cells become interconnected by plasmodesmata forming a symplastic domain. All these observations are consistent with earlier results (Hoth et al., 2008). The phloem around giant cells responded to auxin throughout the development of the females and even after the cells have assumed sieve element identity. In agreement with the observation that no cells expressing *SUC2* could be found in root knots we hardly ever observed cells that were exclusively responding to auxin without also testing positively for the sieve element marker. This is an additional line of evidence that there are no companion cells in root knots rather than the possibility that the companion cells within this structure are simply not expressing the *SUC2* gene. We showed earlier that the sieve elements in root knots are frequently nucleated and are in principle capable of a transcriptional response to the hormone. We can, however, not rule out that some of these sieve elements developed further and their nuclei degraded. This implies that the GFP is still detectable in a fully differentiated state of the sieve element that exhibits slow or no protein turn over. There are no data on the half life time of GFP in fully developed sieve elements but GFP is a fairly stable protein with a reported half life time of up to 24 h even in rapidly dividing cultures of *E. coli* (Andersen et al., 1998). Taken together the results show that all cells that assumed sieve element identity were experiencing an auxin response. Consistently, an auxin response element is found in the promoter of the sieve element marker whose expression pattern fits perfectly with the phloem symplastic domain surrounding giant cells (Figure 7E). We hypothesize that auxin response is a prerequisite of sieve element identity if formed by secondary processes.

In contrast to auxin we did not observe a cytokinin response mediated by B-type ARRs in root-knots. This does not rule out that there is cytokinin response *per se* in root knots. In an earlier study ARR5 was used to monitor cytokinin response (Lohar et al., 2004). ARR5 was found to be induced in the cells around giant cells. This particular gene belongs to the A-type cytokinin response regulators that repress cytokinin signaling by a negative feedback loop. This suggests that in the sieve elements around giant cells processes activated by cytokinin need to be tightly regulated and are not taking place at an elevated level. It is possible that a negative regulation of downstream targets occurs and perhaps represses further phloem differentiation, i.e., metaphloem development. This is further substantiated by the notion that there appear to be some similarities between giant cell vascularization and lateral root formation, i.e., the expression of A-type ARR genes and the lack of response to cytokinin as judged by the expression of B-type ARRs while, at the same time, an high auxin responsiveness is observed (Benkova and Bielach, 2010; Bishopp et al., 2011a). Our data obtained from the infection of auxin responsive mutants are pointing toward the same direction. In agreement with other studies where the same mutants were infected with *H. schachtii*, the infection rates were low. In marked contrast, lateral root formation obviously occurred frequently, especially associated with the root knots (Goverse et al., 2000). A possible explanation is that once the feeding site is successfully initiated the vascularization occurs independently of these particular *AXR* genes or gene combinations. Due to the high auxin response around the giant cells even in the mutant background in combination with high A-type ARR and low B-type ARR response the ratio of auxin to cytokinin enables lateral root formation at almost normal levels. Nevertheless, further studies involving cytokinin resistant mutants are necessary to gain insight in the role of this hormone.

Our results raise the question what the identity of the phloem in root knots is. It is possible that the phloem is secondary metaphloem. This would imply that in root knots metaphloem initials produce only sieve element daughters and that the auxin/cytokinin balance plays a role in this process. While we cannot rule out this possibility and clearly, more data are needed to come to a definite answer there are several lines of evidence that favor a different hypothesis. Obviously, this phloem shares certain obvious features with protophloem i.e., the cells are nucleated frequently and we never detected companion cells, a cell type that is generally found in metaphloem. The fact that we found only very few cells in a mature root knot, which exclusively displayed an auxin response as we observed in fully differentiated metaphloem companion cells, as well as the absence of SUC2 points to the notion that companion cells are really absent from root knots. It remains elusive how and why companion cells are consumed or absent from the phloem in root knots. A possible explanation is that the sieve elements display a mixed identity. Physiologically, the repression of *SUC2* or not expressing the gene makes a lot of sense. Assimilates, including sucrose, need to leave the unloading phloem and be sequestered in the apoplast. The presence of SUC2 would lead to re-import of sucrose into the phloem. Turning down the expression of SUC2 would direct the flow of sucrose into giant cells, provided they possess a means to facilitate the uptake of sucrose. Indeed earlier studies found another sucrose transporter, SUC1 to be specifically up-regulated in root knots (Hammes et al., 2005). The possibility that the sieve elements in root knots are protophloem is supported by the expression of the *APL* gene in the phloem of root knots. This gene encodes a transcription factor that is required to specify phloem cells. In the wild type root it is expressed in the protophloem sieve elements and in the metaphloem in companion cells (Bonke et al., 2003). However, since APL is also expressed in early metaphloem sieve elements (Helariutta, personal communication), the true nature of these sieve elements remains elusive. The fact that companion cells are absent from root knots clearly favors the possibility that the sieve elements are protophloem sieve elements. Furthermore, protophloem is induced in wol/fass double mutants, suggesting that protophloem sieve elements do not require a cytokinin response (Scheres et al., 1995). As stated above there are certain similarities between lateral root formation and root knot vascularization. It is important to notice that also during lateral root formation the first phloem cells, which are formed and connect the new organ to the existing vasculature, are protophloem cells. Taken together our results indicate that the differential auxin and cytokinin response that occurs in the cells around giant cells would induce them to proliferate and assume protophloem identity. This would require at least one more signal in addition to auxin and cytokinin that triggers protophloem rather than metaphloem development and keeps the protophloem in an active state for a relatively long period of time. Obviously, this would also imply that there are no signals that trigger development of metaphloem in root knots or alternatively a mechanism is active that suppresses metaphloem differentiation in root knots.

#### Syncytia

As previously published the phloem around syncytia consists of sieve elements and companion cells as it has been shown in a number of studies using molecular markers suggesting it is typical metaphloem (Hoth et al., 2005, 2008). We found that both cell types, sieve elements and companion cells responded to auxin whereas only sieve elements but not companion cells responded to cytokinin in a B-type ARR mediated fashion. In the case of companion cells the responsiveness to both hormones was also observed in the wild type roots of a similar age, i.e., they responded to auxin, but not to cytokinin, indicating that these are general features of metaphloem companion cells. On the other hand the fact that we observed an auxin and cytokinin response in the sieve elements seems surprising because typically fully differentiated sieve elements lack nuclei and, therefore, cannot respond on a transcriptional level. In marked contrast to the situation in root knots the phloem elements surrounding syncytia still display the typical elongated shape and we therefore assumed that the sieve elements would be typical metaphloem sieve elements. However, it should be kept in mind that the phloem around syncytia is of secondary origin and proliferates to support the development of the feeding site. It is possible that some of the sieve elements still contained nuclei and the degeneration of the nucleus had not already taken place. The metaphloem sieve elements may have performed an auxin response shortly after the division of the metaphloem initial and the GFP is still detectable as explained above. It is known that the metaphloem proliferates around syncytia but there are significantly more sieve elements than companion cells produced (Hoth et al., 2005). It is also well-known that cytokinins are required for secondary growth and proliferation of vascular elements in the shoots (Matsumoto-Kitano et al., 2008; Nieminen et al., 2008; Hejatko et al., 2009). It is tempting to speculate that the ratio of auxin to cytokinin response in the two cell types has an impact on the number of cell divisions the cells undergo after the initial division of the metaphloem initial. If the ratio is in favor of cytokinin the sieve elements proliferate and in the opposite case the cells assume companion cell identity. This implies that high auxin levels in companion cells restrict their proliferation and may contribute to companion cell identity. It will be interesting to find out if similar processes hold true in the limited secondary growth of the Arabidopsis root and to find out why auxin and cytokinin act exclusively on phloem proliferation in the vicinity of nematode-induced feeding sites.

## Conclusion

The data presented here suggest that the phloem around giant cells is protophloem but the phloem around syncytia is metaphloem. This indicates that the ratio of auxin and cytokinin triggers the development and proliferation of different types of phloem. More markers for proto- and metaphloem are needed to address the question how the auxin/cytokinin balance causes different effects on the development of these tissues. These effects can—so far—only be explained by the existence of a signal secreted from the feeding sites that acts in combination with auxin and cytokinin and induces the proliferation of different types of phloem and also suppresses xylem development.

### Conflict of interest statement

The authors declare that the research was conducted in the absence of any commercial or financial relationships that could be construed as a potential conflict of interest.
